# Bis(tetra­methyl­ammonium) thio­sulfate tetra­hydrate

**DOI:** 10.1107/S1600536811021672

**Published:** 2011-06-18

**Authors:** Yun-Xia Yang, Seik Weng Ng

**Affiliations:** a Key Laboratory of Polymer Materials of Gansu Province, Ministry of Education, College of Chemistry and Chemical Engineering, Northwest Normal University, Lanzhou 730070, Gansu, People’s Republic of China; b Department of Chemistry, University of Malaya, 50603 Kuala Lumpur, Malaysia

## Abstract

The anion of the title salt, 2C_4_H_12_N^+^·S_2_O_3_^2−^·4H_2_O, possesses approximate *C*_3v_ symmetry. The water mol­ecules themselves engage in hydrogen bonding, forming a ribbon running along the *a* axis; adjacent chains are linked to the thio­sulfate anions by hydrogen bonds, forming a three-dimensional network. The cavities in the network are occupied by the tetra­methyl­ammonium counter ions.

## Related literature

For tetra­ethyl­ammonium thio­sulfate dihydrate, see: Leyten *et al.* (1988 ▶).
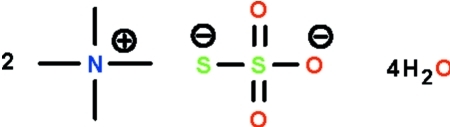


## Experimental

### 

#### Crystal data

2C_4_H_12_N^+^·S_2_O_3_^2−^·4H_2_O
*M* *_r_* = 332.48
Monoclinic, 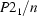

*a* = 8.1869 (1) Å
*b* = 15.4342 (2) Å
*c* = 14.0867 (2) Å
β = 94.074 (1)°
*V* = 1775.47 (4) Å^3^
*Z* = 4
Mo *K*α radiation
μ = 0.33 mm^−1^
*T* = 130 K
0.25 × 0.20 × 0.15 mm

#### Data collection

Bruker SMART APEX diffractometer
Absorption correction: multi-scan (*SADABS*; Sheldrick, 1996 ▶) *T*_min_ = 0.923, *T*_max_ = 0.953
11725 measured reflections
4080 independent reflections
3531 reflections with *I* > 2σ(*I*)
*R*_int_ = 0.019

#### Refinement

*R*[*F* ^2^ > 2σ(*F* ^2^)] = 0.045
*wR*(*F* ^2^) = 0.127
*S* = 1.02
4080 reflections
204 parameters
12 restraints
H atoms treated by a mixture of independent and constrained refinement
Δρ_max_ = 0.74 e Å^−3^
Δρ_min_ = −0.26 e Å^−3^

### 

Data collection: *APEX2* (Bruker, 2007 ▶); cell refinement: *SAINT* (Bruker, 2007 ▶); data reduction: *SAINT*; program(s) used to solve structure: *SHELXS97* (Sheldrick, 2008 ▶); program(s) used to refine structure: *SHELXL97* (Sheldrick, 2008 ▶); molecular graphics: *X-SEED* (Barbour, 2001 ▶); software used to prepare material for publication: *publCIF* (Westrip, 2010 ▶).

## Supplementary Material

Crystal structure: contains datablock(s) global, I. DOI: 10.1107/S1600536811021672/xu5240sup1.cif

Structure factors: contains datablock(s) I. DOI: 10.1107/S1600536811021672/xu5240Isup2.hkl

Supplementary material file. DOI: 10.1107/S1600536811021672/xu5240Isup3.cml

Additional supplementary materials:  crystallographic information; 3D view; checkCIF report

## Figures and Tables

**Table 1:** Hydrogen-bond geometry (Å, °)

*D*—H⋯*A*	*D*—H	H⋯*A*	*D*⋯*A*	*D*—H⋯*A*
O1*W*—H11⋯O1	0.83 (1)	1.88 (1)	2.706 (3)	174 (3)
O1*W*—H12⋯O3*W*	0.84 (2)	1.92 (2)	2.758 (2)	178 (1)
O2*W*—H21⋯O1*W*	0.84 (2)	1.86 (2)	2.689 (2)	172 (2)
O2*W*—H22⋯O4*W*	0.84 (2)	1.90 (3)	2.736 (2)	173 (3)
O3*W*—H31⋯O2^i^	0.83 (2)	1.93 (2)	2.759 (2)	172 (2)
O3*W*—H32⋯O2*W*^ii^	0.84 (2)	1.88 (2)	2.713 (2)	173 (2)
O4*W*—H41⋯S2^iii^	0.83 (2)	2.51 (2)	3.3280 (19)	170 (2)
O4*W*—H42⋯O3*W*^iii^	0.83 (2)	1.93 (2)	2.760 (2)	179 (2)

Symmetry codes: (i) 
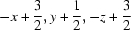
; (ii) 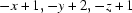; (iii) 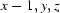.

**Fig. 1.**
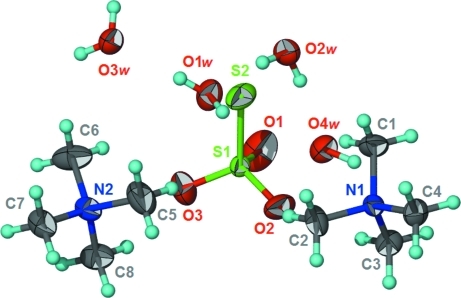
Thermal ellipsoid plot (Barbour, 2001) of 2(CH3)4N+ S2O32-.4H2O at the 70% probability level; hydrogen atoms are drawn as spheres of arbitrary radius.

**Fig. 2.**
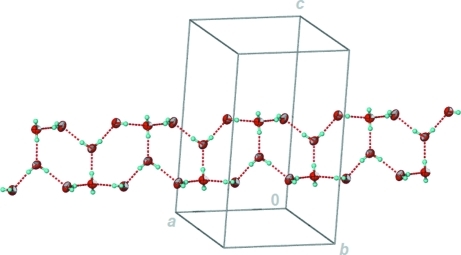
Ribbon motif arising from hydrogen bonds involving water molecules.

## supplementary crystallographic information

### Comment

The thiosulfate anion of tetramethylammonium thiosulfate tetrahydrate (Scheme I, Fig. 1) resulted from the decomposition of 1,2-hydrazinedicarbothioamide under basic conditions. The anion of the salt, tetramethylammonium thiosulfate tetrahydrate, possesses approximate *C*_3v_ symmetry. The four water molecules themselves engage in hydrogen bonding to form a ribbon running along the *a*-axis of the monoclinic unit cell; adjacent chains are linked to the thiosulfate anion by hydrogen bonds to form a three-dimensional network. The cavities in the network are occupied by the ammonium counterions. Tetraethylammonium thiosulfate exists as a dihydrate; in this salt, the sulfur-sulfur bond is 2.028 (1) Å (Leyten *et al.*, 1988).

### Experimental

1,2-Hydrazinedicarbothioamide (0.25 mmol, 0.038 g) was dissolved in tetramethylammonium hydroxide (25% aqueous solution) in a 1:2 molar ratio. A small quantity of water-ethanol (1:2) was added to dissolve the reactants completely. The mixture was set aside for the growth of colorless crystals, which separated after several days.

### Refinement

Carbon-bound H-atoms were placed in calculated positions (C—H 0.98 Å) and were included in the refinement in the riding model approximation, with *U*(H) set to 1.5*U*(C).

The water H-atoms were located in a difference Fourier map, and were refined with a distance restraint of O–H 0.84±0.01 Å; their temperature factors were freely refined.

### Crystal data

**Table:**

2C_4_H_12_N^+^·S_2_O_3_^2^^−^·4H_2_O	*F*(000) = 728
*M**_r_* = 332.48	*D*_x_ = 1.244 Mg m^−^^3^
Monoclinic, *P*2_1_/*n*	Mo *K*α radiation, λ = 0.71073 Å
Hall symbol: -P 2yn	Cell parameters from 5675 reflections
*a* = 8.1869 (1) Å	θ = 2.8–27.6°
*b* = 15.4342 (2) Å	µ = 0.33 mm^−^^1^
*c* = 14.0867 (2) Å	*T* = 130 K
β = 94.074 (1)°	Block, colorless
*V* = 1775.47 (4) Å^3^	0.25 × 0.20 × 0.15 mm
*Z* = 4	

### Data collection

**Table:**

Bruker SMART APEX diffractometer	4080 independent reflections
Radiation source: fine-focus sealed tube	3531 reflections with *I* > 2σ(*I*)
graphite	*R*_int_ = 0.019
ω scans	θ_max_ = 27.6°, θ_min_ = 2.0°
Absorption correction: multi-scan (*SADABS*; Sheldrick, 1996)	*h* = −10→10
*T*_min_ = 0.923, *T*_max_ = 0.953	*k* = −20→20
11725 measured reflections	*l* = −18→12

### Refinement

**Table:**

Refinement on *F*^2^	Primary atom site location: structure-invariant direct methods
Least-squares matrix: full	Secondary atom site location: difference Fourier map
*R*[*F*^2^ > 2σ(*F*^2^)] = 0.045	Hydrogen site location: inferred from neighbouring sites
*wR*(*F*^2^) = 0.127	H atoms treated by a mixture of independent and constrained refinement
*S* = 1.02	*w* = 1/[σ^2^(*F*_o_^2^) + (0.0501*P*)^2^ + 2.4726*P*] where *P* = (*F*_o_^2^ + 2*F*_c_^2^)/3
4080 reflections	(Δ/σ)_max_ = 0.001
204 parameters	Δρ_max_ = 0.74 e Å^−^^3^
12 restraints	Δρ_min_ = −0.26 e Å^−^^3^

### Fractional atomic coordinates and isotropic or equivalent isotropic displacement parameters (Å^2^)

**Table:**

	*x*	*y*	*z*	*U*_iso_*/*U*_eq_	
S1	0.69577 (6)	0.66503 (3)	0.67106 (3)	0.02068 (13)	
S2	0.83493 (9)	0.67111 (4)	0.56042 (5)	0.04154 (18)	
O1	0.5466 (2)	0.71568 (13)	0.64692 (18)	0.0556 (6)	
O2	0.6474 (3)	0.57509 (11)	0.68608 (12)	0.0437 (5)	
O3	0.7837 (3)	0.70123 (13)	0.75481 (13)	0.0495 (5)	
O1W	0.4917 (2)	0.88844 (12)	0.63615 (14)	0.0384 (4)	
O2W	0.2208 (2)	0.92082 (12)	0.52239 (12)	0.0346 (4)	
O3W	0.7668 (2)	0.99207 (10)	0.64424 (12)	0.0322 (4)	
O4W	−0.0083 (2)	0.85939 (12)	0.63760 (13)	0.0353 (4)	
N1	0.7309 (2)	0.37242 (11)	0.56622 (11)	0.0223 (3)	
N2	1.2319 (2)	0.64015 (13)	0.83568 (12)	0.0277 (4)	
C1	0.7226 (3)	0.43773 (15)	0.48818 (16)	0.0361 (5)	
H1A	0.7122	0.4958	0.5152	0.054*	
H1B	0.6274	0.4257	0.4440	0.054*	
H1C	0.8226	0.4346	0.4540	0.054*	
C2	0.8740 (3)	0.39114 (17)	0.63428 (16)	0.0344 (5)	
H2A	0.8628	0.4494	0.6608	0.052*	
H2B	0.9749	0.3880	0.6010	0.052*	
H2C	0.8785	0.3484	0.6859	0.052*	
C3	0.5774 (3)	0.37662 (15)	0.61729 (16)	0.0301 (5)	
H3A	0.5661	0.4346	0.6444	0.045*	
H3B	0.5823	0.3335	0.6685	0.045*	
H3C	0.4831	0.3645	0.5725	0.045*	
C4	0.7481 (3)	0.28336 (14)	0.52585 (16)	0.0309 (5)	
H4A	0.6541	0.2711	0.4808	0.046*	
H4B	0.7520	0.2407	0.5775	0.046*	
H4C	0.8492	0.2800	0.4928	0.046*	
C5	1.2059 (4)	0.5696 (2)	0.76359 (19)	0.0487 (7)	
H5A	1.2113	0.5132	0.7957	0.073*	
H5B	1.2911	0.5727	0.7183	0.073*	
H5C	1.0981	0.5766	0.7295	0.073*	
C6	1.2232 (3)	0.72610 (19)	0.7873 (2)	0.0452 (7)	
H6A	1.1151	0.7332	0.7536	0.068*	
H6B	1.3079	0.7295	0.7417	0.068*	
H6C	1.2407	0.7721	0.8349	0.068*	
C7	1.3963 (3)	0.62940 (17)	0.88745 (16)	0.0337 (5)	
H7A	1.4021	0.5728	0.9189	0.051*	
H7B	1.4132	0.6753	0.9353	0.051*	
H7C	1.4815	0.6331	0.8421	0.051*	
C8	1.1031 (3)	0.63532 (18)	0.90535 (17)	0.0374 (5)	
H8A	1.1080	0.5786	0.9368	0.056*	
H8B	0.9950	0.6431	0.8719	0.056*	
H8C	1.1217	0.6811	0.9532	0.056*	
H11	0.515 (3)	0.8363 (7)	0.641 (2)	0.036 (8)*	
H12	0.576 (2)	0.9191 (13)	0.638 (2)	0.058 (10)*	
H21	0.3104 (18)	0.912 (2)	0.5532 (18)	0.046 (8)*	
H22	0.145 (2)	0.903 (3)	0.554 (2)	0.085 (14)*	
H31	0.791 (4)	1.0218 (15)	0.6926 (11)	0.049 (9)*	
H32	0.768 (5)	1.0224 (17)	0.5952 (11)	0.076 (12)*	
H41	−0.053 (3)	0.8120 (9)	0.625 (2)	0.053 (9)*	
H42	−0.077 (3)	0.8988 (12)	0.640 (2)	0.055 (10)*	

### Atomic displacement parameters (Å^2^)

**Table:**

	*U*^11^	*U*^22^	*U*^33^	*U*^12^	*U*^13^	*U*^23^
S1	0.0216 (2)	0.0191 (2)	0.0214 (2)	−0.00144 (18)	0.00176 (17)	−0.00134 (17)
S2	0.0512 (4)	0.0395 (3)	0.0368 (3)	−0.0140 (3)	0.0236 (3)	−0.0079 (3)
O1	0.0277 (10)	0.0471 (11)	0.0919 (16)	0.0091 (8)	0.0034 (10)	0.0030 (11)
O2	0.0689 (13)	0.0263 (8)	0.0374 (9)	−0.0122 (8)	0.0146 (9)	0.0000 (7)
O3	0.0599 (13)	0.0547 (12)	0.0332 (9)	−0.0133 (10)	−0.0017 (9)	−0.0143 (8)
O1W	0.0280 (9)	0.0380 (10)	0.0478 (10)	−0.0030 (8)	−0.0066 (7)	0.0038 (8)
O2W	0.0292 (9)	0.0409 (9)	0.0332 (8)	−0.0024 (7)	−0.0026 (7)	0.0054 (7)
O3W	0.0383 (9)	0.0256 (8)	0.0327 (8)	−0.0050 (7)	0.0021 (7)	−0.0009 (7)
O4W	0.0325 (9)	0.0321 (9)	0.0413 (9)	−0.0017 (7)	0.0022 (7)	0.0032 (7)
N1	0.0268 (9)	0.0203 (8)	0.0198 (8)	−0.0010 (7)	0.0009 (6)	−0.0012 (6)
N2	0.0214 (9)	0.0385 (10)	0.0233 (8)	0.0043 (8)	0.0016 (7)	0.0029 (7)
C1	0.0504 (15)	0.0282 (11)	0.0295 (11)	−0.0031 (10)	0.0016 (10)	0.0086 (9)
C2	0.0273 (11)	0.0459 (13)	0.0294 (11)	−0.0015 (10)	−0.0028 (9)	−0.0093 (10)
C3	0.0276 (11)	0.0300 (11)	0.0334 (11)	0.0010 (9)	0.0073 (9)	−0.0009 (9)
C4	0.0390 (13)	0.0214 (10)	0.0326 (11)	−0.0012 (9)	0.0045 (9)	−0.0062 (8)
C5	0.0539 (17)	0.0598 (18)	0.0321 (12)	−0.0035 (14)	0.0000 (11)	−0.0122 (12)
C6	0.0370 (14)	0.0514 (16)	0.0482 (15)	0.0112 (12)	0.0110 (11)	0.0238 (13)
C7	0.0226 (11)	0.0459 (13)	0.0321 (11)	0.0077 (10)	−0.0022 (9)	0.0019 (10)
C8	0.0278 (12)	0.0495 (14)	0.0364 (12)	0.0050 (10)	0.0116 (9)	0.0074 (11)

### Geometric parameters (Å, °)

**Table:**

S1—O3	1.4496 (18)	C1—H1C	0.9800
S1—O2	1.4631 (17)	C2—H2A	0.9800
S1—O1	1.4696 (19)	C2—H2B	0.9800
S1—S2	1.9970 (8)	C2—H2C	0.9800
O1W—H11	0.828 (9)	C3—H3A	0.9800
O1W—H12	0.834 (10)	C3—H3B	0.9800
O2W—H21	0.837 (10)	C3—H3C	0.9800
O2W—H22	0.841 (10)	C4—H4A	0.9800
O3W—H31	0.834 (10)	C4—H4B	0.9800
O3W—H32	0.835 (10)	C4—H4C	0.9800
O4W—H41	0.831 (10)	C5—H5A	0.9800
O4W—H42	0.832 (10)	C5—H5B	0.9800
N1—C2	1.488 (3)	C5—H5C	0.9800
N1—C1	1.489 (3)	C6—H6A	0.9800
N1—C3	1.493 (3)	C6—H6B	0.9800
N1—C4	1.498 (3)	C6—H6C	0.9800
N2—C6	1.490 (3)	C7—H7A	0.9800
N2—C8	1.493 (3)	C7—H7B	0.9800
N2—C7	1.494 (3)	C7—H7C	0.9800
N2—C5	1.494 (3)	C8—H8A	0.9800
C1—H1A	0.9800	C8—H8B	0.9800
C1—H1B	0.9800	C8—H8C	0.9800
			
O3—S1—O2	111.83 (11)	N1—C3—H3B	109.5
O3—S1—O1	109.87 (13)	H3A—C3—H3B	109.5
O2—S1—O1	108.00 (12)	N1—C3—H3C	109.5
O3—S1—S2	109.83 (9)	H3A—C3—H3C	109.5
O2—S1—S2	109.44 (8)	H3B—C3—H3C	109.5
O1—S1—S2	107.78 (10)	N1—C4—H4A	109.5
H11—O1W—H12	111.2 (16)	N1—C4—H4B	109.5
H21—O2W—H22	108.9 (16)	H4A—C4—H4B	109.5
H31—O3W—H32	110.5 (16)	N1—C4—H4C	109.5
H41—O4W—H42	111.3 (16)	H4A—C4—H4C	109.5
C2—N1—C1	109.68 (18)	H4B—C4—H4C	109.5
C2—N1—C3	109.39 (16)	N2—C5—H5A	109.5
C1—N1—C3	109.27 (17)	N2—C5—H5B	109.5
C2—N1—C4	109.40 (17)	H5A—C5—H5B	109.5
C1—N1—C4	109.96 (16)	N2—C5—H5C	109.5
C3—N1—C4	109.11 (17)	H5A—C5—H5C	109.5
C6—N2—C8	109.37 (19)	H5B—C5—H5C	109.5
C6—N2—C7	109.53 (19)	N2—C6—H6A	109.5
C8—N2—C7	109.12 (17)	N2—C6—H6B	109.5
C6—N2—C5	109.8 (2)	H6A—C6—H6B	109.5
C8—N2—C5	109.7 (2)	N2—C6—H6C	109.5
C7—N2—C5	109.33 (19)	H6A—C6—H6C	109.5
N1—C1—H1A	109.5	H6B—C6—H6C	109.5
N1—C1—H1B	109.5	N2—C7—H7A	109.5
H1A—C1—H1B	109.5	N2—C7—H7B	109.5
N1—C1—H1C	109.5	H7A—C7—H7B	109.5
H1A—C1—H1C	109.5	N2—C7—H7C	109.5
H1B—C1—H1C	109.5	H7A—C7—H7C	109.5
N1—C2—H2A	109.5	H7B—C7—H7C	109.5
N1—C2—H2B	109.5	N2—C8—H8A	109.5
H2A—C2—H2B	109.5	N2—C8—H8B	109.5
N1—C2—H2C	109.5	H8A—C8—H8B	109.5
H2A—C2—H2C	109.5	N2—C8—H8C	109.5
H2B—C2—H2C	109.5	H8A—C8—H8C	109.5
N1—C3—H3A	109.5	H8B—C8—H8C	109.5

### Hydrogen-bond geometry (Å, °)

**Table:**

*D*—H···*A*	*D*—H	H···*A*	*D*···*A*	*D*—H···*A*
O1W—H11···O1	0.83 (1)	1.88 (1)	2.706 (3)	174 (3)
O1W—H12···O3W	0.84 (2)	1.92 (2)	2.758 (2)	178.(1)
O2W—H21···O1W	0.84 (2)	1.86 (2)	2.689 (2)	172 (2)
O2W—H22···O4W	0.84 (2)	1.90 (3)	2.736 (2)	173 (3)
O3W—H31···O2^i^	0.83 (2)	1.93 (2)	2.759 (2)	172 (2)
O3W—H32···O2W^ii^	0.84 (2)	1.88 (2)	2.713 (2)	173 (2)
O4W—H41···S2^iii^	0.83 (2)	2.51 (2)	3.3280 (19)	170 (2)
O4W—H42···O3W^iii^	0.83 (2)	1.93 (2)	2.760 (2)	179 (2)

Symmetry codes: (i) −*x*+3/2, *y*+1/2, −*z*+3/2; (ii) −*x*+1, −*y*+2, −*z*+1; (iii) *x*−1, *y*, *z*.
